# Insights into the miRNA regulations in human disease genes

**DOI:** 10.1186/1471-2164-15-1010

**Published:** 2014-11-21

**Authors:** Jyotirmoy Das, Soumita Podder, Tapash Chandra Ghosh

**Affiliations:** Bioinformatics Centre, Bose Institute, P 1/12, C.I.T. Scheme VII M, Kolkata, 700 054 India

**Keywords:** MicroRNAs, Cancer, mRNA decay rates, AREScores, Epigenetic modifications

## Abstract

**Background:**

MicroRNAs are a class of short non-coding RNAs derived from either cellular or viral transcripts that act post-transcriptionally to regulate mRNA stability and translation. In recent days, increasing numbers of miRNAs have been shown to be involved in the development and progression of a variety of diseases. We, therefore, intend to enumerate miRNA targets in several known disease classes to explore the degree of miRNA regulations on them which is unexplored till date.

**Results:**

Here, we noticed that miRNA hits in cancer genes are remarkably higher than other diseases in human. Our observation suggests that UTRs and the transcript length of cancer related genes have a significant contribution in higher susceptibility to miRNA regulation. Moreover, gene duplication, mRNA stability, AREScores and evolutionary rate were likely to have implications for more miRNA targeting on cancer genes. Consequently, the regression analysis have confirmed that the AREScores plays most important role in detecting miRNA targets on disease genes. Interestingly, we observed that epigenetic modifications like CpG methylation and histone modification are less effective than miRNA regulations in controlling the gene expression of cancer genes.

**Conclusions:**

The intrinsic properties of cancer genes studied here, for higher miRNA targeting will enhance the knowledge on cancer gene regulation.

**Electronic supplementary material:**

The online version of this article (doi:10.1186/1471-2164-15-1010) contains supplementary material, which is available to authorized users.

## Background

MicroRNAs (miRNAs) are abundant classes of endogenous small non-coding RNAs approximately 21–23 nucleotides (nts) long transcripts generated from 70–100 nts hairpin precursors, which regulate gene expression post-transcriptionally by affecting the translation of target messenger RNAs (mRNAs) [1]. These small miRNA molecules play important roles in cell growth, differentiation, proliferation, apoptosis, and polarization of neurons [2]. With the recent advancement of experimental studies, many biological factors have been revealed to contribute in the recognition of miRNA targeting [3]. In-depth characterization of miRNA targets enables better understanding of the role of miRNAs in various biological processes.

There is a growing body of evidence regarding the importance of miRNAs in human diseases [4]. Studies have established that mutations, dysregulations or dysfunction of miRNA biogenesis and their targets led to the blockage of physiological and biochemical pathways that influenced various diseases in human [5]. Computational prediction did not only reveal a number of miRNA-disease associations but also showed that the mechanistic associations in miRNAs and human diseases are very complicated. Previously, it has also been demonstrated that genetic defects in miRNAs, their processing machinery and epigenetic regulations are common hallmarks of human diseases [3].

Although a number of studies have dealt with the miRNA-disease association, but no study has yet been conducted in concerning the type of disease class which is more susceptible to miRNA target. In the present study, we aimed to identify the disease class which is more prone to miRNA target. Our studies indicated that the cancer disease genes are more prevalent in miRNA-targeted human disease genes compared to the other disease gene classes. These findings raise the obvious question, why cancer genes are more targeted by miRNAs. To explain this, we have analyzed the role of various gene subcomponents, mRNA decay rates, mRNA stability, AREScores. Finally, we revealed a remarkable insight that the miRNAs have more influence than epigenetic modifications in controlling the expression of cancer related genes.

## Results

### miRNA targets of disease genes

**Figure 1:**
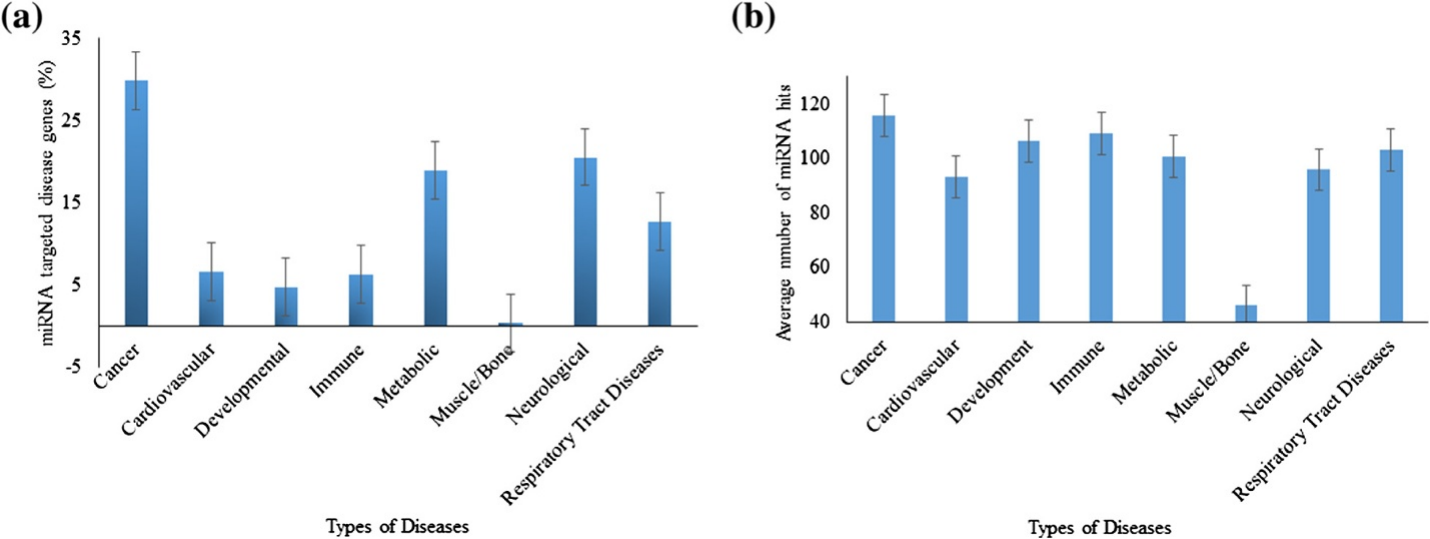
**(a) Distribution of miRNA targeted disease genes (in percentage) (Error bar indicates 5% standard error). (b)** Average number of miRNA hits among cancer and non-cancer disease genes (Error bar indicates 5% standard error).

We have separated human disease genes depending on the miRNA target to the disease genes in two groups: miRNA-targeted disease genes and miRNA non-targeted disease genes. We aimed to find out the disease class which is mostly targeted by miRNA among all disease classes. Thus, we have broadly categorized a total of 301 miRNA targeted disease genes into eight different disease classes according to the Human Gene Mutation Database (HGMD) [6], such as cancer disease genes, neurological disease genes, developmental disease genes, metabolic disease genes, respiratory tract disease genes, immunological disease genes, cardiovascular disease genes and muscle/bone disease genes. The miRNA targeted disease genes category and their corresponding miRNA targets are listed in the online supplement (Additional file 1: Table S1). Measuring the miRNA target profile across the disease gene classes, we have noticed significant differences in their distributions (Figure 1). Among all the disease classes, cancer genes (29.80%) were observed to be mostly targeted by miRNA (Figure 1a). Moreover, we have noticed the average number of miRNA target sites were also higher for cancer genes compared to the other classes of genes (Figure 1b). To exemplify the novel characteristics of cancer genes required for mRNA::miRNA base-pairing, we have merged the other seven disease classes into non-cancer disease group and performed the rest of the study by comparing cancer (N = 90) and non-cancer disease genes (N = 211) (Additional file 1: Table S1).

### Gene subcomponents in cancer genes

It was evident that miRNAs perform their regulatory roles mainly by base-pairing with 3′-UTRs of target genes. Therefore, genes targeted by miRNAs should have longer 3′-UTR sequences [7]. Hence, we have calculated lengths of 3′-UTRs and observed that the average 3′-UTRs length are longer for cancer genes than non-cancer disease genes (Table 1). To determine the effect of other gene subcomponents, we have calculated the length of 5′-UTRs and transcript of these two disease groups since it was ascertained that miRNA also targets at these regions [8]. We have found that both 5′-UTRs lengths and transcript lengths are significantly higher on average for cancer disease genes compared to non-cancer disease genes (Table 1). To understand the relationship between gene subcomponents and number of miRNA hits, we performed correlation analysis [7, 9]. Considering all the disease genes together, we have noticed that both the 3′- and 5′-UTRs as well as transcript length hold significant positive correlations with the number of miRNA hits (*ρ*_3′-UTR vs. miRNA hits_ = 0.532; *P =* 2.2 × 10^−19^, *N* = 243; *ρ*_5′-UTR vs. miRNA hits_ = 0.153; *P =* 4.9 × 10^−2^, *N* = 212; *ρ*_transcript length vs. miRNA hits_ = 0.166; *P =* 4.2 × 10^−2^, *N* = 150).

**Table 1:** **Length of gene structures in miRNA-targeted cancer genes and non-cancer disease genes**

Structural parameters	miRNA-targeted cancer disease genes (bp)	miRNA-targeted non-cancer disease genes (bp)	Level of significance ( ***P***)
Average 3′-UTR lengths	1143	908	4.4 × 10^−2^
Average 5′-UTR lengths	349	254	4.2 × 10^−2^
Average transcript lengths	4196	3418	2.8 × 10^−2^

Therefore, it could be expected that longer structure of genes is most likely to have more complex regulation. Indeed, we have found that longer gene structures (Table 1) is significantly higher in cancer genes than non-cancer disease genes, indicating that gene lengths are more crucial for miRNA regulation of cancer genes (see Additional file 2: Table S2).

### Intrinsic genomic properties of cancer genes

To reveal the pattern of gene expression level of the miRNA-targeted cancer genes, we computed the correlation between miRNA hits and gene expression levels of cancer and non-cancer disease genes separately. We obtained a significant negative correlation between miRNA hits and the expression levels of cancer disease genes *ρ*_expression levels vs. miRNA hits_ = −0.266; *P =* 3.8 × 10^−2^; *N* = 51). However, no significant correlation exists in the case of non-cancer disease genes (*ρ*_expression levels vs. miRNA hits_ = 0.004; *P =* 9.9 × 10^−1^; *N* = 122). Moreover, we have noticed that the percentage of lowly expressed genes are much higher in miRNA-targeted cancer disease genes than non-cancer disease genes (in cancer: 49.18%; in non-cancer: 31.97%; at 95% significance level) which suggests that miRNAs prefer the lowly expressed genes for their target. This observation was also echoed in an earlier report that proposed highly expressed genes could not be targeted by miRNA due to their shorter genomic structure [7].

Next, to examine whether the higher expression of onco-miRNAs repressed the expression of onco-mRNAs, we did correlation analysis between miRNA expression and mRNA expression of cancer genes. We have obtained a negative correlation between them (*ρ*_*mRNA expression* vs. miRNA expression_ = −0.273; *P =* 3.9 × 10^−2^; *N* = 51) (Additional file 3: Figure S1). This result clearly indicates that miRNA plays a significant role in lowering the expression of cancer genes.

Conrad *et al*. [10] showed that duplicate genes play important roles in human diseases and it has also been reported that miRNA is prerequisite for dosage compensation of duplicated genes [11]. Thus, we have tested the prevalence of gene duplications in miRNA-targeted cancer and non-cancer genes and observed that gene duplication are more frequent in cancer genes than non-cancer genes (duplicate frequency in cancer: 58.24%; in non-cancer: 45.02%; at 95% significance level, *N* = 301) (Additional file 3: Figure S2).

Gene duplication is the key regulatory mechanism of genome and organism evolution. It was already reported that genes having more distinct miRNA binding sites at the 3′UTR regions tend to have slower evolutionary rates [9]. Measuring evolutionary rates of the cancer and non-cancer genes, we have noticed that evolutionary rates are slower for miRNA-targeted cancer genes than non-cancer genes (in cancer: 0.1786; in non-cancer: 0.3129; *P =* 3.0 × 10^−6^, *N* = 281) (Additional file 3: Figure S3).

Our observation is in accordance with the report of Thomas *et al*. (2003) [12]. Thus, higher purifying selection on cancer genes could be treated as a trademark of higher miRNA targets.

### Mode of cancer genes regulation by miRNA

Generally, in a cellular environment, miRNAs mediate gene regulation by reducing the stability of their target mRNAs through mRNA degradation methods or using translational repression processes [7]. Thus, we hypothesized that the cancer genes should have more mRNA decay rates than others. Therefore, to test mRNA stability differences between miRNA-targeted cancer disease genes and miRNA-targeted non-cancer genes on the genomic scale, we have compared mRNA decay rates which were taken from Pai *et al.*[13]. We have noticed a significant positive correlation between miRNA targets and mRNA decay rates (*ρ*_miRNA hits vs. mRNA decay rates_ = 0.119; *P* = 5.0 x 10^−2^; *N* = 264) (Additional file 3: Figure S4). This result suggests that the number of miRNA targets may enhance mRNA decay rates which is in agreement with the previous findings that miRNA targeted genes have higher decay rates [7].

Jing *et al.*[14] showed that the ARE-(AU-rich elements) containing mRNA degradation required to be targeted by miRNAs. Therefore, we have calculated the correlation between miRNA targets and AREScore and found a positive correlation between them (*ρ*_miRNA hits vs. AREScore_ = 0.124; *P* = 4.0 x 10^−2^; *N* = 273) (Additional file 3: Figure S5). Higher miRNA targets of cancer genes may be the artifact of higher AU-rich element in cancer genes. Thus, we proposed that cancer genes regulation mediated through miRNA is achieved via lowering the stability of corresponding mRNA.

### Relative contribution of the factors in determining miRNA targets on disease genes

We have studied several factors that might influence the miRNA targets on disease genes. In this section we summarize the relative contribution of each of these factors that contribute significantly for determining miRNA targets on genes. Since, gene duplication data is a binary variable, we have converted rest of the factors into the binary variables and performed logistic regression analysis by taking number of miRNA hits as a dependent variable and gene subcomponents, gene duplication, evolutionary rate, mRNA decay rate, AU-rich elements (AREScores) as the independent variables. We did not consider epigenetic modifications since they did not show any significant effects on miRNA hits in correlation analysis. The result delineated in Table 2 strongly advocates that AREScores is the most important predictor of miRNA hits. The relative contribution of the factors is in the order of AREScores > 3′-UTR lengths > transcript lengths > 5′-UTR lengths.

**Table 2:** **The relative contributions of different parameters on number of miRNA targets**

Variables	β value	Level of significance
ARE Scores	2.712	2.5 × 10^−4^
3′-UTR lengths	1.914	3.4 × 10^−3^
Transcript lengths	1.695	1.0 × 10^−2^
5′-UTR lengths	1.562	3.4 × 10^−2^
Intron lengths	0.335	7.1 × 10^−1^
CDS lengths	−0.090	9.0 × 10^−1^
Gene lengths	−0.151	8.3 × 10^−1^
Paralogs	−0.157	8.4 × 10^−1^
mRNA decay rates	−0.287	6.4 × 10^−1^
Evolutionary Rates	−0.939	1.6 × 10^−1^

### miRNA regulations vs. epigenetic modifications of cancer genes

Proper orchestration of gene expression primarily depends on the epigenetic modifications and miRNA regulation [15]. A close association between epigenetic modifications and miRNAs is just beginning to be understood and has a great importance in the field of molecular biology. Recent studies suggested that miRNAs are considered as the important players in the regulation of epigenetic modifications, such as DNA methylations and histone modifications [16]. We, therefore, intended to investigate whether epigenetic modifications or miRNA plays the most important role in regulating cancer genes expression. For this purpose, we have measured two important epigenetic modifications like CpG methylation and histone modifications in cancer and non-cancer disease genes and observed that the cancer genes have relatively lower level of methylation (68.13%) as compared to non-cancer disease genes (82.94%) (Significant at 99% level, McCullum Proportion test). We did not find any significant correlation (_ρmiRNA hits vs. CpG methylation_ = 0.115, *P* = 7.7 × 10^−1^, *N* = 237) between miRNA hits and CpG methylation which suggests that gene regulation by miRNA and CpG methylation is not interconnected. Histone modifications are another part of epigenetic silencing mechanism in mammals. It was previously reported that non-coding RNAs can direct the cytosine methylation and histone modifications that are related to gene expression regulation in complex organisms among several other unrelated functions [17]. Thereafter, we have computed the histone modification to determine its effect on cancer and non-cancer genes. However, we did not obtain any significant difference in the rate of histone modification between cancer and non-cancer disease genes (in cancer: 677.8118; in non-cancer: 693.5784; *P =* 9.8 × 10^−1^, *N* = 289). Here also we did not get any significant relation between miRNA hits and histone modification (*ρ*_miRNA hits vs. histone modification_ = 0.088, *P* = 1.4 × 10^−1^, *N* = 289).

To analyze the effective roles of miRNA hits and epigenetic modifications on disease gene expression, we performed a partial correlation analysis by considering these factors (i.e., gene expression, miRNA hits, histone modifications, and DNA methylation) and found that disease gene expression is negatively correlated with miRNA hits when both epigenetic modifiers i.e. CpG methylation and histone modifications are controlled (Table 3), but the correlation between gene expression and CpG methylation disappeared when we controlled the miRNA hits and histone modifications. Moreover, we also did not find any significant correlation between gene expression and histone modifications after controlling CpG methylation and miRNA hits (Table 3).

**Table 3:** **Partial correlation analysis for miRNA-targeted cancer gene expression with miRNA hits, CpG methylation and histone modifications**

Factors	Partial correlation for disease gene expression	Level of Significance
Number of miRNA hits	**−0.345** (controlling CpG methylation ratio and histone modification rate)	1.6 × 10^−2^
CpG methylation ratio	**0.169** (controlling histone modification and miRNA hits)	2.5 × 10^−1^
Histone modification rate	**0.143** (controlling miRNA hits and CpG methylation ratio)	3.3 × 10^−1^

These results suggest that miRNAs play more significant role in controlling the disease gene expression than epigenetic modifications such as CpG methylations or histone modifications.

## Discussion

In this manuscript, we have sought to address three questions: First, which class of disease genes is most targeted by miRNAs? Second, why is this particular class of disease genes frequently targeted by miRNAs? Third, whether miRNAs or epigenetic modifications is the major guiding factor in controlling the expression of disease genes in human? We have detected that miRNAs commonly targets the cancer disease genes. To explore the underlying aims of this observation, we have analyzed several structural and functional parameters. We have traced the important role of 3′-UTRs among other gene subcomponents in determining the miRNA target site in cancer disease genes. miRNAs usually target genes which have long 3′-UTRs, and cancer genes are observed to possess longer UTRs than non-cancer disease genes. Therefore, it is quite reasonable that miRNA readily target the cancer disease genes. We also tested gene expression levels and noticed that lowly expressed genes are abundant in miRNA targeted cancer genes. Consequently, we also observed that miRNA targeted cancer genes are evolutionary slower than miRNA targeted non-cancer disease genes. Since miRNA targeted genes are proposed to be evolutionary slower [7], the slower evolutionary rate of cancer genes could be treated as a good substrate for miRNA target compared to non-cancer disease genes. However, the relation between expression and evolutionary rate pursued in our study may arise a controversy since evolutionarily conserved genes are evident to have higher expression levels [18]. However, it is also reported that highly expressed genes are generally linked with lower mRNA decay rates, and genes undergoing rapid mRNA decay, are enriched with putative binding sites for miRNA and RNA binding proteins [13]. In our case, we observed that the cancer genes have higher mRNA decay rates due to higher AU-rich elements and miRNA target sites. So, higher mRNA decay rates may bring down the expression of miRNA targeted cancer genes. Moreover, the reduction of mRNA levels by miRNAs in cancer genes also suggested the reasonable hypothesis that miRNAs in some cases could stimulate mRNA decay through increasing decapping rates [19]. The longer gene structure of poorly expressed miRNA targeted cancer genes also supports the ‘selection for economy’ theory that explains the highly and broadly expressed genes have to be shorter to reduce the high energy cost for transcription [20]. This observation also implies that the expression of miRNA targeted cancer genes cannot be attributed to their slower evolutionary rate. This could be treated as an exceptional case where expression could not explain the reason for slower evolutionary rates. It may be the selective stringency of miRNA targeted genes since they possess longer 3′-UTR structure which is reported to hold strong negative correlation with evolutionary rates [9], or it may be the integral property of cancer disease genes as they are over-represented in the collection of essential genes [12] which are known to be evolutionarily conserved [21]. In addition, intense purifying selection may help to prevent multigene interactions concerned in certain cancers [12]. Moreover, higher duplicability of miRNA targeted cancer genes observed in our study was also relevant to their slower evolutionary rates.

Now the question remains, miRNA targets or epigenetic modifications which one controls the gene expression in miRNA-targeted cancer disease genes. In our study, we noticed that the epigenetic regulation is less likely to control the expression of cancer genes compared to miRNA regulations. Occasionally, miRNA also gets deregulated by several mechanisms like inefficient processing of miRNAs through drosha or dicer etc. This epigenetic silencing of miRNAs in cancer cells modulates the activity of oncogenes as well as the tumor suppressor genes [22]. So, it is quite natural if miRNA gets deregulated, it will induce disease phenotypes. In our result, we found that cancer disease is more prevalent than non-cancer disease genes in miRNA deregulated disease gene set (average number of miRNA targets: in cancer = 4.0222; in non-cancer = 1.7488, *P* = 1.0 × 10^−14^, *N* = 301). Persistence of human miRNA genes in the genomic regions involved in the loss of heterozygosity, amplification or breakpoints in cancers [18], also suggesting a link between miRNA and the development of cancer.

## Conclusions

In summary, we have reported ample evidences to support the link between longer gene structure, higher enrichment of AREs, higher duplicability, slower evolutionary rates, and lower mRNA decay rates of cancer genes that make them good substrate for miRNA targeting. Regression analysis has established AU-rich elements as the most influential genomic property that determines miRNA hits on disease genes. The cause and fate of miRNA targets on cancer genes analyzed in our study will help in enhancing the knowledge and medicinal improvement of cancer genes.

## Methods

### Human disease genes and microRNA data

Human (*Homo sapiens)* disease genes were collected from The International Cancer Gene Consortium (ICGC, ftp://data.dcc.icgc.org/), Human Gene Mutation Database (HGMD, http://www.hgmd.cf.ac.uk/ac/index.php) [6], and Genetic Association Database (GAD, http://geneticassociationdb.nih.gov/) [23]. Disease genes in our study have been classified in eight major disease classes, like cancer, cardiovascular, developmental, immunological, metabolic, muscle/bone disorder, neurological and respiratory tract diseases according to HGMD.

After removing the redundancy in disease gene datasets, we considered only those genes for which miRNA targets information are available in TargetScan release 6.2 database (http://www.targetscan.org/) [24] and DIANA TarBase v6.0 (http://diana.imis.athena-innovation.gr/DianaTools/index.php) [25]. It was reported that TargetScan have high fidelity for target prediction from biological and informatics validation. TargetScan is used for its reported accuracy and advantages of seed-pairing mechanisms in miRNA (which is required for mRNA-miRNA bindings) over other miRNA databases. Furthermore, to increase the reliability of our results, we only considered the miRNAs whose target sites are conserved across most mammals (as defined by TargetScan) [24]. We used context+score as defined by Garcia *et al*. [26]. DIANA TarBase provides experimentally verified data and it is the largest available manually curated target database. In order to achieve more consistent results, we have considered only those miRNA targets those are present in both the prediction database as well as experimental database. Finally, we have collected only a total of 301 miRNA-targets for our analysis (Additional file 1: Table S1).

### Gene subcomponent data

Gene subcomponents, such as 3′-UTR lengths, 5′-UTR lengths, transcript lengths data were collected from UCSC genome browser (hg19) [27, 28] and Ensembl Biomart database v69 [29] (Additional file 2: Table S2).

### Gene expression data

The mRNA expression data collected from GNF Gene Atlas (http://biogps.org/). The expression values were averaged over tissues and sorted into ascending order. Then the values are equally divided into three groups. The first group containing low expressed genes, the second group is of medium expressed genes and the third one contains highly expressed genes.

Next, we have retrieved miRNA expression data from CancerMiner database [30] which contains microarray expression data for ten tissues of cancer patients. We have averaged the expression values over tissues and then mapped the miRNA expression levels with our dataset (Additional file 2: Table S2).

### Identification of paralogs

Human paralogs were downloaded from ENSEMBL database (v69) [29]. We have collected 148 duplicated genes data after removing the redundant entries and taking the paralogous similarity cut-off value 40% for our data [11] (Additional file 2: Table S2).

### Calculation of evolutionary rate

Protein evolutionary rates [*d*N/*d*S] data for human using 1:1 orthology relationship to Chimpanzee (*Pan troglodytes*) were downloaded from ENSEMBL database (v69). Next, we mapped the evolutionary rate data to miRNA targeted human disease genes for further analysis (Additional file 2: Table S2).

### mRNA decay rate and ARE score data

A total of 16,823 mRNA decay rate data was obtained from Pai *et al.*[13] dataset. We have mapped the data with our dataset and finally collected 267 data for our analysis. AREScore data was collected from AREScore database (http://arescore.dkfz.de/arescore.pl) [31] by assigning all default parameter setup (Additional file 2: Table S2).

### Collection of epigenetic data

Histone modification data were retrieved from human histone modification database (HHMD, http://202.97.205.78/hhmd/index.jsp) [32] which contains useful histone modifications information from experimental data that is essential for understanding the modifications at a systematic level (Additional file 2: Table S2). We have collected DNA methylation data from NGSmethDB (http://bioinfo2.ugr.es/NGSmethDB/index.php) [33] database which is based on next-generation sequencing DNA methylation data from different human tissues (Additional file 2: Table S2).

### Statistical analysis

All statistical analysis except partial correlations was performed using SPSS v20 and R v3.0.2. TANAGRA (v1.4) [34] was used to determine the partial correlation. For correlation analysis, we have used Spearman’s rank correlation test since all of our data shows non-parametric distribution. To test the non-parametric distributions of our dataset we have performed Shapiro-Wilk test (used for the dataset smaller than 2000) (Table 4). To find the difference between two datasets, we have performed Mann–Whitney U test (Two-tailed test). The results are considered to be significant if the P-value is less than 0.05. We used McCullum proportion test to verify the confidence level of the proportion data used in the study.

**Table 4:** **Shapiro-wilk test results to show the non-parametric dataset used in our study**

Parameters	W score	***P***value
ARE Scores	0.786	1.3 × 10^−16^
3′-UTR lengths	0.951	4.4 × 10^−7^
Transcript lengths	0.931	2.0 × 10^−6^
5′-UTR lengths	0.785	2.6 × 10^−15^
Paralogs	0.824	4.5 × 10^−12^
mRNA decay rates	0.735	2.2 × 10^−22^
Evolutionary Rates	0.787	7.4 × 10^−19^
CpG methylation	0.891	4.5 × 10^−12^
Histone modification rate	0.699	2.0 × 10^−22^
miRNA hits	0.929	7.8 × 10^−11^
mRNA expression levels	0.193	8.3 × 10^−27^

## Electronic supplementary material

Additional file 1: Table S1: miRNA targeted disease genes category and their corresponding number and name of miRNA hits. (XLSX 646 KB)

Additional file 2: Table S2: miRNA targeted disease genes and their different characteristics (gene subcomponents, miRNA expression, gene duplication, evolutionary rates, mRNA decay rate, AU rich element, CpG methylation, Histone modifications) analyzed in our study. (XLSX 36 KB)

Additional file 3: Figures S1: Correlation between microRNA expression levels and microRNA targeted repressed mRNA expression levels in Cancer disease genes. **S2.** Percentage of duplicated genes in miRNA-targeted cancer and non-cancer disease genes. **S3.** Mean difference of evolutionary rates of cancer and non-cancer disease genes in human. **S4.** Correlation between mRNA decay rates and number of miRNA targets. **S5.** Correlation between AU-rich element scores and number of miRNA targets. (DOCX 170 KB)
