# Supplementary material for: Insights into the miRNA regulations in human disease genes
Source: BMC Genomics. 2014 Nov 21;15(1):1010. doi: 10.1186/1471-2164-15-1010 (PMC4256923; doi:10.1186/1471-2164-15-1010)

Supplementary Figures:

**Figure S1**: Correlation between microRNA expression levels and microRNA targeted repressed mRNA expression levels in Cancer disease genes.


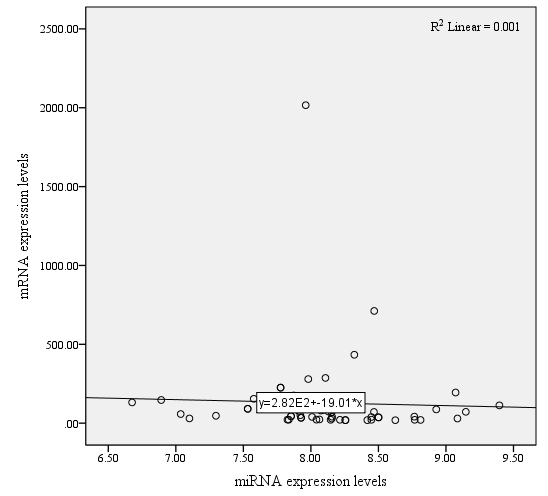


**Figure S2:** Percentage of duplicated genes in miRNA-targeted cancer and non-cancer disease genes.


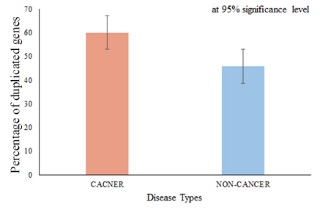


**Figure S3**: Mean difference of evolutionary rates of cancer and non-cancer disease genes in human.


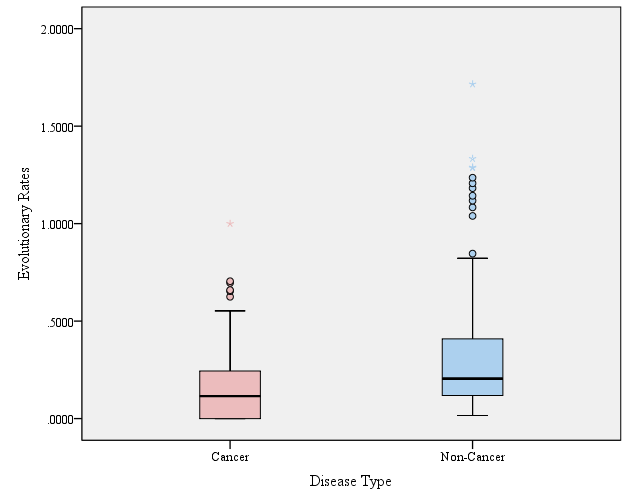


**Figure S4**: Correlation between mRNA decay rates and number of microRNA targets.


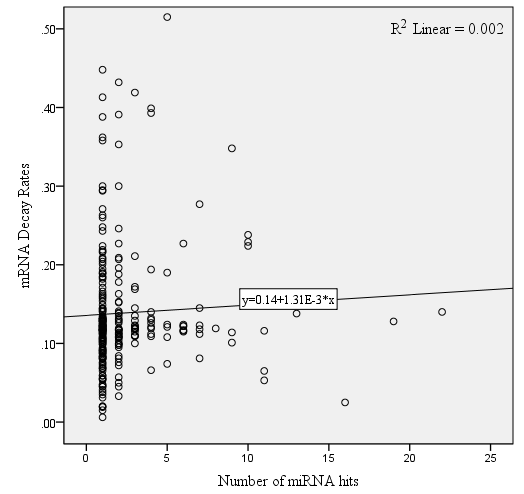


**Figure S5**: Correlation between AU-rich element scores and number of microRNA targets.


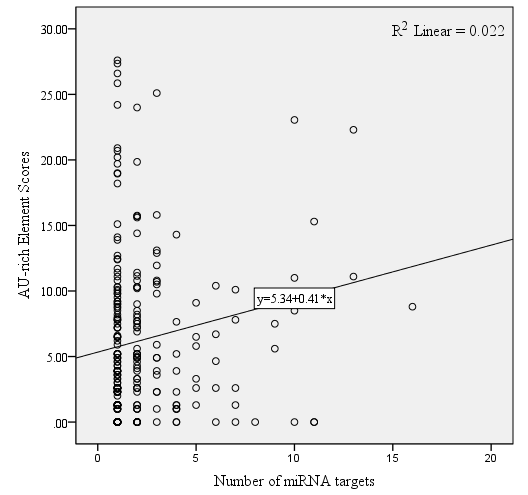

Supplement: Supplementary file 3 — Additional file 3: Figures S1: Correlation between microRNA expression levels and microRNA targeted repressed mRNA expression levels in Cancer disease genes. S2. Percentage of duplicated genes in miRNA-targeted cancer and non-cancer disease genes. S3. Mean difference of evolutionary rates of cancer and non-cancer disease genes in human. S4. Correlation between mRNA decay rates and number of miRNA targets. S5. Correlation between AU-rich element scores and number of miRNA targets. (DOCX 170 KB) [file 12864_2014_6713_MOESM3_ESM.docx]
